# RFID-Based Vehicle Positioning and Its Applications in Connected Vehicles

**DOI:** 10.3390/s140304225

**Published:** 2014-03-04

**Authors:** Jianqiang Wang, Daiheng Ni, Keqiang Li

**Affiliations:** 1 State Key Laboratory of Automotive Safety and Energy, Tsinghua University, Beijing 100084, China; E-Mails: wjqlws@tsinghua.edu.cn (J.W.); likq@tsinghua.edu.cn (K.L.); 2 Department of Civil and Environmental Engineering, University of Massachusetts, Amherst, MA 01003, USA

**Keywords:** RFID, vehicle positioning, connected vehicle technology

## Abstract

This paper proposed an RFID-based vehicle positioning approach to facilitate connected vehicles applications. When a vehicle passes over an RFID tag, the vehicle position is given by the accurate position stored in the tag. At locations without RFID coverage, the vehicle position is estimated from the most recent tag location using a kinematics integration algorithm till updates from the next tag. The accuracy of RFID positioning is verified empirically in two independent ways with one using radar and the other a photoelectric switch. The former is designed to verify whether the dynamic position obtained from RFID tags matches the position measured by radar that is regarded as accurate. The latter aims to verify whether the position estimated from the kinematics integration matches the position obtained from RFID tags. Both means supports the accuracy of RFID-based positioning. As a supplement to GPS which suffers from issues such as inaccuracy and loss of signal, RFID positioning is promising in facilitating connected vehicles applications. Two conceptual applications are provided here with one in vehicle operational control and the other in Level IV intersection control.

## 1. Introduction

On 3 February 2014, The U.S. Department of Transportation's (DOT) officially announced its decision to move forward with vehicle-to-vehicle communication technology for light vehicles. After decade-long research and experimentation, this decision signifies USDOT's resolution to transform transportation safety and mobility by allowing cars to “talk” with each other. A long list of innovative applications have been tested or under the way including cooperative collision warning [1–3] intersection safety supporting [4], intersection movement assist, *etc*. In these applications, real-time vehicle positioning is assumed in their algorithms and protocols for motion guidance, operational control, and interaction with other vehicles. This is a reasonable assumption since Global Positioning System (GPS) technology has become widely available and affordable. As a matter of fact, many vehicles have already been equipped with GPS for navigation and tracking purposes. However, GPS-based vehicle positioning begins to show its limitations as connected vehicles are advancing toward real-world implementation and especially when the success of these applications depends heavily on the accuracy of vehicle positioning. These limitations include poor or no signals in certain areas especially urban canyon, and positioning accuracy in a dynamic environment.

To address the above limitations, this paper proposes a supplementary yet independent approach, *i.e.*, radio-frequency identification (RFID)-based vehicle positioning, to facilitate connected vehicle applications at critical locations where GPS service is unavailable or unreliable.

This paper is arranged as follows: the next section identifies research gaps based on a survey of literature in terms of vehicle positioning technologies. Following that, Section 3 proposes the RFID-based vehicle positioning approach with design details. Section 4 verifies the accuracy of the RFID-based vehicle positioning in two empirical ways. Section 5 provides two conceptual examples to illustrate the application of RFID-based vehicle positioning in connected vehicles. Lastly, conclusions are drawn in Section 6.

## 2. Research Gap in Vehicle Positioning

Due to its wide coverage and availability, GPS seems to be ideal for connected vehicle applications [5,6]. Stand-alone GPS has the capability of achieving an accuracy of about 20–30 m, which can be narrowed down to 8–12 m after the removal of the selective availability [7]. Differential GPS can even enhance accuracy further to 1–2 m. However, it relies on ground-based reference stations which only covers limited areas and, thus, significantly drives up the cost [8].

In connected vehicles, the requirement on vehicle positioning varies with the nature of application. In general, those involving a large spatial and temporal scope, such as roadway incidence assistance and dynamic routing, do not require accurate positioning updated at a high frequency. For example, an accuracy of 5–10 m is required to warn drivers of hazard at a fixed location (e.g., an accident site), to which general purpose GPS receivers suffice the need. In contrast, applications in small spatial and temporal areas, such as motion control especially crash avoidance, require accurate positioning in real time. For example, most safety applications require one to two meters [9] Shladover *et al.* [10] pointed out that assigning vehicles to the correct lanes would require a standard deviation of about 1 m, but 50 cm accuracy is likely to produce significantly better performance especially for blind spot warning. Combined with adverse locations such as urban canyon, these situations pose a great challenge to GPS-based positioning. As such, further enhancements of GPS or alternative vehicle positioning technologies are called for. Consequently, a number of approaches have been proposed including inertial systems, dead reckoning, information fusion, and map matching.

To improve the positioning performance with GPS/DGPS, a common choice is to integrate it with inertial systems. For example, Farrell *et al.* [11] implemented a real-time carrier phase DGPS aided inertial navigation system which is able to achieve an accuracy at the centimeter level. Huang and Tan [12] used a Kalman filter to incorporate in-vehicle motion sensors in the refinement of vehicle position. However, Jiménez *et al.* [13] pointed out that this approach is valid only if inertial measurements are used before DGPS signals begin to degrade. In addition, the fine accuracy relies on DGPS which is a costly solution only available at limited locations. Dead reckoning [14] advances a vehicle's position from its last known position by integrating its speeds over elapsed time and course. However, this approach is only good for a short period of time and is subject to cumulative errors.

Closely related to the use of inertial systems is the fusion of information [15]. For example, GPS signals can be combined with inertial sensors and digital maps to infer the best estimate of vehicle location [16]; Edelmayer *et al.* [17] used a cooperative federated filtering approach to enhance position estimation based on a variety of position measurements, e.g., from on-board vehicle positioning system, from other cooperating vehicles in the vicinity, as well as from the immediate roadside environment via communication. Bevly [18] attempted to correct inertial sensor errors by using a kinematic Kalman filter estimator to integrate GPS signals, accelerometers, and rate gyroscopes. Islam *et al.* [19] implemented a multi sensor system consisting of a single-axis gyroscope and an odometer integrated with GPS receiver. Though information fusion can achieve high accuracy in some cases, the resultant position is inevitably an estimate that depends on multiple sources of information. Error or missing of a component would degrade estimation quality.

Map matching is to determine the position of a vehicle by constructing a trajectory from a few reliable locations that the vehicle has recently passed and then matching this trajectory to a digital map to find the best fit among multiple likely arcs [20,21]. This approach is best suited for applications that rely on a GPS receiver as the sole means of positioning. However, the uncertainty introduced by inferences in the underlying algorithm limits its use in safety-related applications.

Therefore, in order to obtain high reliability, low cost, and sufficient accuracy under all operational conditions, there exists a great demand for alternative approaches that are readily available, do not rely on GPS, and minimize the need for estimation and fusion. In this context, the approach of using radio sensors such as infrared, microwave, and radio frequency devices has received increasing attention [22,23]. Capable of tracking moving objects [24], these devices can be mounted at roadside to transmit and receive data from vehicles passing in close proximity if they are equipped with transceivers. These systems have been employed in several research projects [25–27] and already been used in transportation such as vehicle speed control [28], real-time bus recognition [29], group location management [30], and electronic toll collection. Due to its low cost and reasonable accuracy, radio-frequency identification (RFID) is promising as a supplement to GPS in connected vehicle applications at critical locations where GPS is unavailable or unreliable but the demand for real-time positioning is high. In the next section, we present an RFID-based vehicle positioning approach and two conceptual applications of the above nature are provided in Section 5.

## 3. RFID-Based Vehicle Positioning

**Figure 1.**
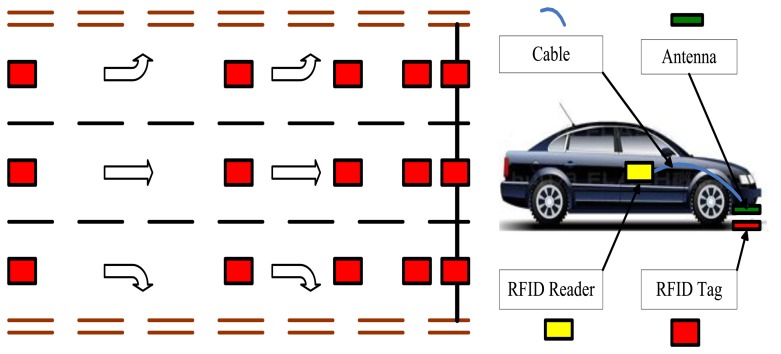
Layout of RFID tags and reader.

**Figure 2.**
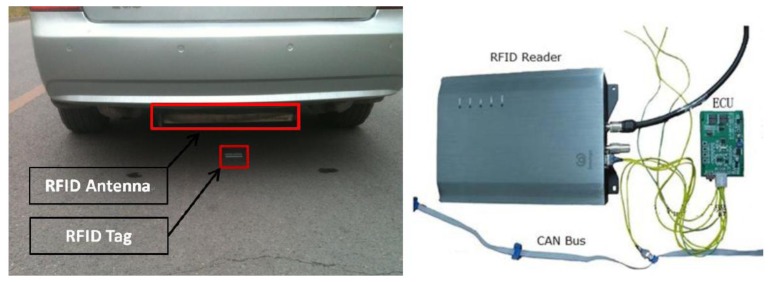
RFID tag, antenna, reader, and controller unit.

RFID tags are a series of passive RFID tags which are fastened on road surface containing position information, e.g., the distance to a reference point, lane number, and direction of travel. When a vehicle passes above an RFID tags, the RFID reader and antenna carried by the vehicle activates the tag and reads in the position information. The layout of the RFID position system is illustrated in Figure 1 and hardware installation pictured in Figure 2. An example design of the format of position information is provided in Table 1.

In this setup, we used an XCAF-12L Panel Antenna (Invengo Information Technology, Co. Ltd., Shenzen, China) which is a rugged UHF directional antenna with a central frequency of 915MHz and circular polarization. The RFID Reader was an Invengo XCRF-502E with a working frequency of 902–928 MHz and working range up to 10 meters. The RFID Tags were ZT-T80s with an effective range of 2–100 m and identification speed up to 200 km/h.

**Figure 3.**
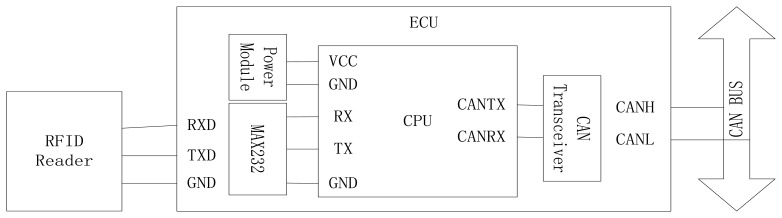
The connection of RFID reader and ECU.

To facilitate the communication between the reader and the tags, an electronic control unit (ECU) was developed. As indicated in Figure 2, the ECU was used to control the reader by RS232 and to transfer data to other modules by CAN Bus. The connection of RFID reader, ECU and CAN Bus is shown in Figure 3. The ECU includes a power module and CPU is based on the Motorola 9s08DZ16 chip. The serial port transceiver is a MAX232 and the CAN transceiver is a TJA1050.

**Figure 4.**
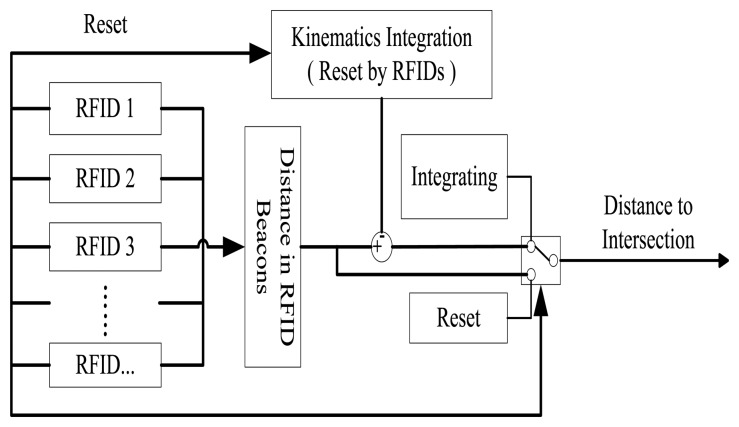
Kinematic integration algorithm.

Since each RFID tags contains static position information at a fixed location, a need arises for a vehicle in motion to acquire its accurate position in a continuous fashion in order to support connected vehicle applications. As such, a kinematics integration algorithm has been devised and added to the RFID positioning system, see Figure 4.

The algorithm calculates vehicle position as follows:

$$\begin{array}{l} d_{2}=d_{\text{tag}}+d_{1}\\ d_{1}=\{\begin{array}{ll} 0 & F_{\text{tag}}=1\\ \underset{k}{\text{∑}}v_{k}\tau +\frac{1}{2}\underset{k}{\text{∑}}a_{k}\tau^{2} & F_{\text{tag}}=0 \end{array} \end{array}$$

where, *d*_2_ is current position; *d*_tag_ is the stored position obtained from RFID tag last time; *d*_1_ is estimated driving distance according to speed integral; *F*_tag_ is a flag whose value is 1 (the system is able to read information from RFID tag) or 0 (otherwise); *k* is data sequence number, starting to count when the system fails to read the tag and reset to 0 when reading resumes; *v* and *a* are vehicle speed and acceleration respectively; *τ* is time elapsed since last successful reading from RFID tag.

## 4. Experimental Verification

The accuracy of the RFID positioning system can be affected by RFID communication range and distance between tags. Since RFID only communicates within a few meters, reading from a tag only occurs when a vehicle moves over the tag, which ensures accuracy. If the vehicle fails to obtain position updates from tags, its position has to be estimated. The longer the kinematics integration runs, the larger the estimation error. Therefore, it is necessary to avoid long gaps between tags to ensure accuracy. In order to verify the feasibility and the accuracy of the positioning approach, this paper proposes two test methods with one based on radar and the other photoelectric switch.

### 4.1. Experimental Verification Based on Radar

**Figure 5.**
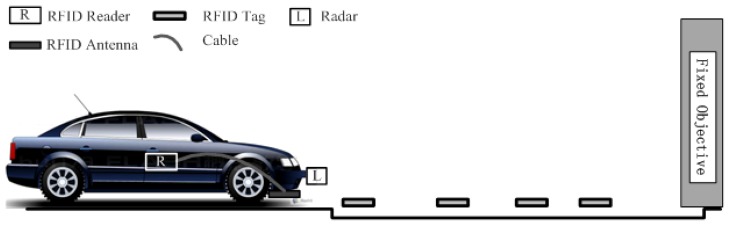
Setup of experimental verification based on radar.

The objective is to verify whether the dynamic position obtained from the tags matches the “true” position of the vehicle measured by the radar. The experiment is set up as shown in Figure 5. The experiment vehicle is equipped with radar, RFID reader and its antenna. The radar is installed on the vehicle's front fender guard. The radar wave beam is oriented forward in the direction of travel. The antenna is installed below the fender guard, and the surface of the antenna senses the ground. The tags are installed on the test road, at the end of which is a fixed target to help radar measure distance. The radar features a millimeter wave with frequency 76∼77 GHz, range up to 180 m, and resolution 0.7 m.

**Figure 6.**
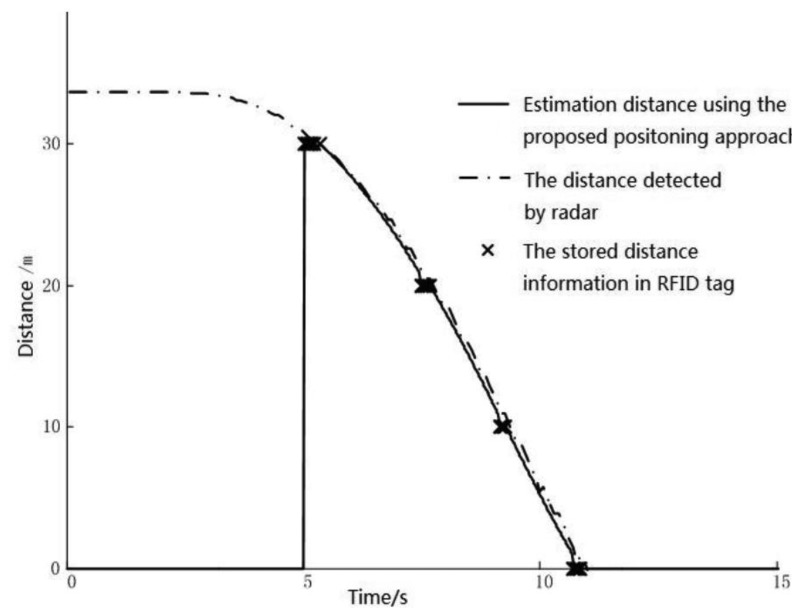
Results of experimental verification based on radar.

In the experiment, the vehicle passes through each tag consecutively while the vehicle accelerates and decelerates several times. The computer on board calculates the distance between the vehicle and the last tag using the proposed approach. The radar measures the distance between the vehicle and the fixed target independently. The results obtained from these two methods are all transferred to the CAN bus, which can be logged in the computer. The comparison of the test results is shown in Figure 6. Note that the estimated distance is zero at the beginning since there is no tag reading and hence nothing to estimate. Starting from the 5th second, tag readings become available and vehicle position estimation begins. The result shows that positions from radar, tags, and estimation match very well.

### 4.2. Experimental Verification Based on Photoelectric Switch

**Figure 7.**
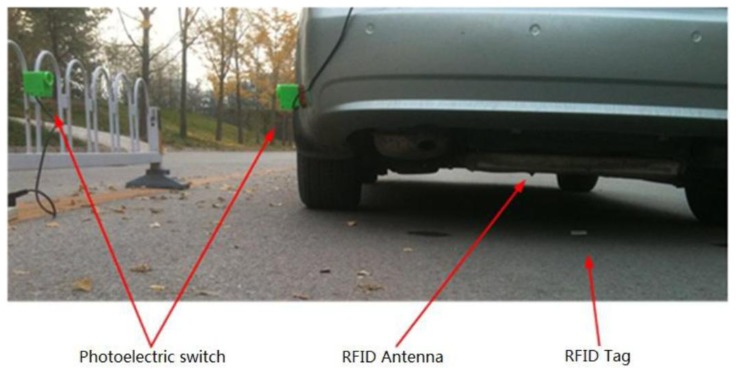
Setup of experimental verification based on photoelectric switch.

The objective focuses on verifying whether the position estimated from kinematics integration matches the position obtained from the tags. The experiment is set up as shown in Figure 7.

The photoelectric switch consists of a transmitter which is fixed at roadside and a receiver which is fixed at the outside of the vehicle. Make sure that the transmitter is in the same cross section as a tag, while the receiver is also in the same cross section as the RFID antenna. As such, when the receiver moves with the vehicle and is aligned with the transmitter, both the RFID and the switch are triggered simultaneously. Starting from this instant, the on-board computer begins to estimate vehicle position using kinematics integration. Meanwhile, another source of position information is obtained from RFID tags.

**Figure 8.**
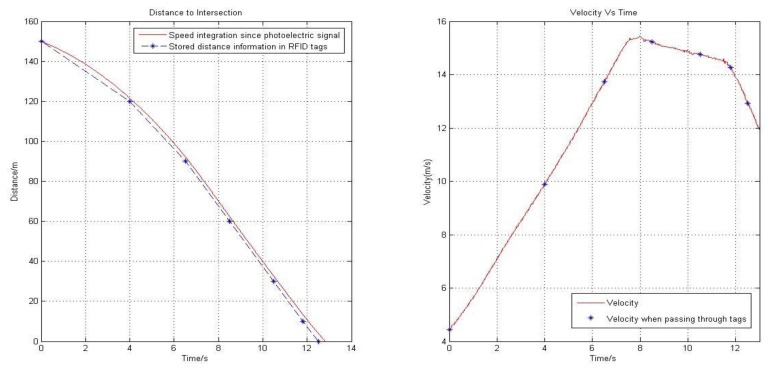
Results of experimental verification based on photoelectric switch.

Figure 8 shows the result of one of the tests. In this test, the error of position is about 5.4% in the first 30 m probably due to accelerating; when speed is relative stable, the errors drops to around 2.5%. It is also noticeable that, as the estimation goes on, the accumulated error increases. Further tests with lower maximum speed (e.g., 36 km/h) reduced the above errors to 3.1% and 1.8% respectively.

The error in position is mainly derived from the accumulation error caused by velocity inaccuracy, especially when the vehicle is accelerating or decelerating. Accordingly, a calibration algorithm is derived using least square method:

$$\begin{array}{c} \Delta d=h_{1}+h_{2}a\\ d_{c}=\Delta d+d_{1} \end{array}$$

where Δ*d* is the position error; *d_c_* is the calibrated position based on integral, *a* is the vehicle acceleration. The coefficients are estimated as *h*_1_ = −1.79 and *h*_2_ = 0.0613. After calibration, the errors in the first test drop to 0.07% and 0.66% respectively.

Limited by time and resources, this research only conducted the above simple, straightforward tests. Nevertheless, the test results revealed that the proposed RFID is promising in providing accurate vehicle positioning in a dynamic process. Before large-scale applications, it is suggested that further tests be performed in more realistic environment (e.g., involving multiple lanes and mixed traffic) with better knowledge of ground truth.

## 5. Example Applications in Connected Vehicles

Allowing vehicle-to-vehicle and vehicle-to-infrastructure communication, connected vehicle technology opens the door to many innovative applications such as intelligent cruise control [28] that transform safety and throughput. Presented below are two conceptual paradigms in which the above RFID positioning approach helps achieve the goals of connected vehicle technology.

### 5.1. Vehicle Operational Control

**Figure 9.**
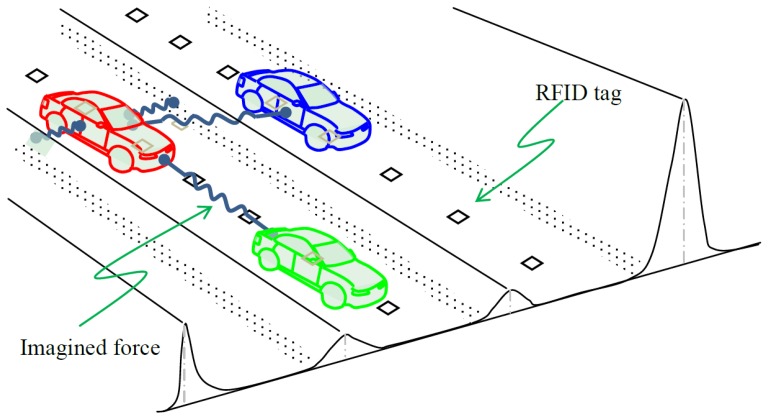
Application of RFID positioning in operational control of connected vehicles.

With accurate information about positions and speeds of connected vehicles, it is feasible to synchronize these vehicles on one or more special, managed lanes at high speeds without compromising safety. Such a paradigm is illustrated in Figure 9. RFID tags on the ground pinpoint the location of each vehicle which is equipped with a cooperative driving assistance system.

To ensure safety in the lateral direction, deviation from lane center is translated to a potential field [31,32] that vehicle *i* needs to overcome. This potential field $U_{i}^{y}$ is imagined in the lateral direction *y* as bumps along the lane lines, road edge, and center line. By taking the first derivative of $U_{i}^{y}$ with respective to *y*, one obtains the correction force *N_i_* that is necessary to steer the vehicle back on track:

$$N_{i}=\partial U_{i}^{y}/\partial y$$

where *N_i_* is imagined as the spring between vehicle *i* and lane line bump, and *N_i_* can be implemented in the actuator that controls vehicle steering. Still in the lateral direction, vehicle *k* in the vicinity poses a safety hazard. As a result, driver *i* may choose to “shy” away and this effect becomes more remarkable when *k* is a heavy truck. Similar to the treatment of lane deviation, the mechanism to avoid parallel running can be created by imagining a repulsive force $F_{i}^{k}$ which is illustrated as the spring between vehicles *i* and *k*. Such a force can be derived from the potential field of *k* perceived by *i*, $U_{i}^{k}$:

$$F_{i}^{k}=\partial U_{i}^{k}/\partial y$$

To ensure safety in the longitudinal direction, a mechanism to maintain safe car following is essential. Hence, the safety hazard in the longitudinal direction *x* can be represented as a potential field $U_{i}^{j}$ of the leading vehicle *j* perceived by *i*. Therefore, the repulsive force $F_{i}^{j}$ that imposes *i* to keep safe distance can be generically derived from $U_{i}^{j}$ as above, a more concrete form of which can be found in [33]:

$${\ddot{x}}_{i}=A_{i}\left|1-\left(\frac{{\dot{x}}_{i}}{v_{i}}\right)-e^{1-\left(\frac{s_{ij}}{s_{ij}^{*}}\right)}\right|$$

where *ẍ_i_* is the operational control (acceleration or deceleration) of vehicle *i*, *A_i_* is the maximum acceleration desired by driver *i* when starting from standing still, *ẋ_i_* is the speed of vehicle *i*, *v_i_* the desired speed of driver *i*, *s_ij_* is the actual spacing between vehicle *i* and its leading vehicle *j*, and $s_{ij}^{*}$ is the desired value of *s_ij_*.

### 5.2. Level IV Intersection Control

An intersection is a point in transportation systems where two or more streams of traffic meet and share roadway capacity. To ensure traffic safety, three levels of intersection control are used conventionally. Level I control does not use any physical device to assign priority to traffic, but rather it relies on each driver understanding and observing basic rules specified in Driver's Manuals such as yielding to vehicles on the right and vehicles already in the intersection. If safety hazard poses an issue (typically identified through intersection sight triangle analysis [34,35]), Level II control may be considered which implements YIELD and/or STOP signs to resolve conflict [36]. Currently, the ultimate form of intersection control is Level III, *i.e.*, intersection signalization [36] which alternately assigns right-of-way to specific movements through signal indication such as Green, Yellow, and Red.

Though potentially capable of reducing certain types of crash, Level III control may give rise to other types of collision and negatively impact efficiency. For example, pre-timed signal control ignores the dynamics of approaching traffic, so green time may be wasted on approaches with light or no traffic; Even though actuated control is made traffic-aware, it is not flexible enough to accommodate demands with varying patterns, especially issues caused by unnecessary calls, mandatory minimum green, and arbitrary max out.

Interestingly, the above three levels of control seems to be no match in many aspects to the old-fashioned traffic control by a police officer. For example, the officer is able to watch vehicle clearing an intersection before releasing traffic from a conflicting approach. By clearing before releasing, conflicting vehicles are well protected. In addition, waste of time is minimized since right-of-way is switched right after clearance. For another example, the officer has full flexibility to assign a relatively long green time to an approach to match its demand or to skip this approach if there is no demand. Moreover, the officer may optimize traffic heuristically on a cycle-by-cycle basis to achieve the overall success of competing objectives such as safety, throughput, and reducing delay.

**Figure 10.**
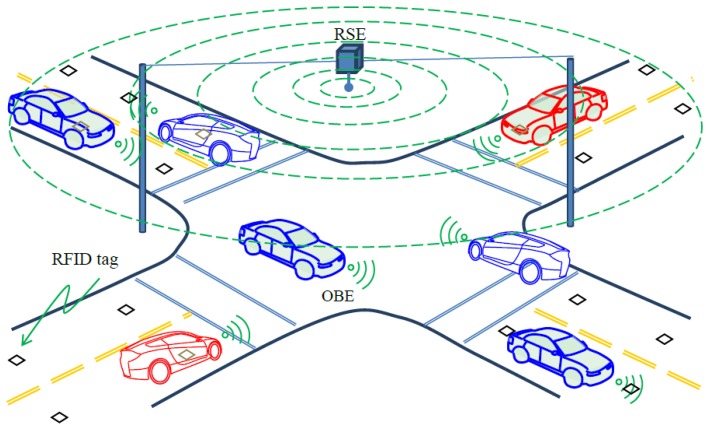
Level IV Intersection control.

The only drawback of this officer-directing-traffic paradigm is that it requires the presence of a trained officer around the clock which is impractical. Fortunately, the advent of connected vehicles, combined with sound positioning technology, makes it possible to reproduce this safe yet efficient paradigm electronically which can be called Level IV control. Figure 10 illustrates such a paradigm where each vehicle is able to talk to other vehicles through on-board equipment (OBE) and communicate with the roadside equipment (RSE) at the intersection. The RFID positioning can help by providing real-time, accurate vehicle positions and speeds, with which the RSE can serve as an “electronic police officer” to direct traffic. More specifically, the RSE can send individualized instruction to each driver regarding stop/go and travel speed. Within the RSE, the internal logic dynamically optimizes traffic based on current demands and vehicle positions, resolves conflict, issues customized command to each driver, monitor vehicle status, and update instructions accordingly. Note that the above discussion concerns only about technical feasibility of Level IV control without complicating the problem with legal and moral issues.

## 6. Conclusions

This paper proposes an RFID approach as a helpful alternative to positioning in connected vehicle applications where GPS is not available or of poor quality. This approach installs RFID tags on the road surface and on-board tag readers in vehicles. When a reader passes over a tag, the reader can receive the position information stored in the tag. To fill gaps between tags, estimation has to be made based on the latest position update from tags. As such, a kinematics integration method is proposed to serve the purpose. When vehicles accelerate or decelerate, their speeds are changing, which affects the accuracy of the estimation method. Error of this nature can be diminished by applying the proposed calibration algorithm.

Road experiments are carried out to validate the RFID-based positioning approach. One type of experiments involves both radar and RFID reader on board. The radar is used to provide “true” positions of the test vehicle, against which estimates from RFID-based positioning are compared. The result shows good match between the two sources of vehicle positions. The other type of experiments focuses on verifying whether the position estimated from the kinematics integration matches the position obtained from the tags. A photoelectric switch is used to trigger the estimation of vehicle position based on the latest tag update. The results indicate that the error of position is about 5.4% during acceleration or deceleration process and around 2.5% when speed is relative stable. With the help of calibration algorithm, the errors can drop to 0.07% and 0.66% respectively. Before large-scale applications, further tests are recommended in more realistic environment with better knowledge of ground truth.

RFID-based positioning appears promising in connected vehicle applications due to its low cost and reasonable accuracy. Two conceptual applications are conceived in this paper. One application deals with vehicle operation control where RFID position provides accurate vehicle positions to enable the prediction of safety hazard. The other application conceives a Level IV intersection control where RFID position makes it possible to conduct traffic by an “electronic police officer”.

## Figures and Tables

**Table 1.** Definition of the data stored in RFID tags.

**Bit**	**Length**	**Value Range**	**Note**
0	1	0	Start Bit
1	1	0, 1	Stop Sign: 1: Yes; 0: No
2∼9	8	0×00∼0×FF	Distance to Stop sign on intersection, Unit: m
10∼12	3	0×0∼0×7	Lane No.: 0: Go Straight; 1: Turn Left
13∼14	2	0∼3	2: Turn Right; 3: U-Turn; 4: Go Straight and Turn Left; 5: Go Straight and Turn Right; 6: All Directions
15	1	0, 1	Orientation: 0: East; 1: South;
